# Macronutrient content of pooled donor human milk before and after Holder pasteurization

**DOI:** 10.1186/s12887-019-1427-5

**Published:** 2019-02-12

**Authors:** Pasqua Piemontese, Domenica Mallardi, Nadia Liotto, Chiara Tabasso, Camilla Menis, Michela Perrone, Paola Roggero, Fabio Mosca

**Affiliations:** 1 NICU Fondazione IRCCS Ca’ Granda Ospedale Maggiore Policlinico, Milan, Italy; 2 Department of Clinical Sciences and Community Health, University of Milan, Milan, Italy

**Keywords:** Donor human milk, Pasteurization, Tailored fortification, Macronutrients

## Abstract

**Background:**

Donor human milk (DHM) is the best alternative for preterm infants when their own mother’s milk is unavailable. DHM should be pasteurized to guarantee microbiological safety; however, this process can influence the macronutrient content.

The aim of this study was to investigate the effect of Holder pasteurization (HoP) on DHM macronutrient content.

**Methods:**

Protein, lactose, lipids (g/100 ml) and energy (kcal/100 ml) of DHM pools were analysed before and after HoP (62.5 °C for 30 min) using mid-infrared spectroscopy (HM analyser Miris AB®). The mean macronutrient content before and after HoP was compared by paired t-test. The percentage decreases (Delta%) were calculated.

**Results:**

The change in macronutrient content of 460 pools was determined. Protein, lipids and lactose decreased significantly after HoP (0.88 ± 0.20 vs 0.86 ± 0.20 and 2.91 ± 0.89 vs 2.75 ± 0.84 and 7.19 ± 0.41 vs 7.11 ± 0.48 respectively). The Delta% values were − 2.51 ± 13.12, − 4.79 ± 9.47 and − 0.92 ± 5.92 for protein, lipids and lactose, respectively (*p* ≤ 0.001).

**Conclusion:**

This study confirms that the macronutrient content of DHM, especially in terms of lipids and protein, is reduced after HoP. Therefore, in order to perform a tailored fortification of DHM, the clinicians need to be aware of the somewhat diminished nutrient content of DHM.

## Background

Human milk is widely recognized as the optimal feeding for newborn infants, especially for preterm infants, because of its various benefits in term of immunological, gastrointestinal and neurodevelopmental functions [1].

When their own mothers’ milk is unavailable or not sufficient to satisfy the requirements of preterm babies, donor human milk (DHM) is the best alternative for these vulnerable infants [2].

Data indicate that donor human milk, especially when associated with own mothers’ milk, contributes to reducing the incidence of necrotizing enterocolitis [3], late-onset sepsis [4] and bronchopulmonary dysplasia [5] and improves feeding tolerance [6], compared to formula feeding.

DHM should be pasteurized to guarantee its microbiological safety: Holder pasteurization (HoP) performed at 62.5 °C for 30 min is recommended in the international human milk bank guidelines [7–9]. At the authors’ institution, DHM is used to feed preterm infants (birth weight < 1500 g) or term infants affected by growth restriction (birth weight < to the 3rd percentile of Fenton 2013 cards), pulmonary bronchodysplasia, sepsis, congenital and acquired surgical pathology (omphalocele, gastroschisis, diaphragmatic hernia, volvulus, intestinal perforations, necrotizing enterocolitis), severe intestinal absorption deficit (short post-surgical or functional bowel), severe heart disease, neurological diseases and chromosomopathies, chronic renal failure and immunodeficiencies. Although it is well recognized that pasteurized milk maintains some of the beneficial and protective effects of mother’s milk, it is also known that the heating process used for the treatment of DHM could lead to decreased content of nutrients and bioactive compounds [10].

Considering the requirements of low-birth-weight infants in terms of macronutrients and energy intake, both mother’s milk and DHM should be fortified to assure optimal growth during the first months of life [11]. Several studies have been performed to investigate the effects of HoP on the properties of DHM. For example, in a recent study using 30 samples of pooled DHM, Adhisivam et al. demonstrated that HoP decreases protein, fat and energy content; however, carbohydrates were not significantly reduced [12]. Actually O’Connor et al. [13] and Peila et al. [14] reviewed the impact of collection, storage and pasteurization on the nutritional aspects of human milk [13], however, there is an evident lack of agreement likely due to the heterogeneity of the performed test protocols and the low number of analysed pools [14].

The aim of this study was to investigate the effect of HoP on the DHM macronutrient content.

## Methods

The macronutrient content of pooled DHM of the Human Milk Bank at the authors’ institution was determined before and after HoP. All donors signed a written informed consent to donate their milk to clinical or research use therefore the ethics committee of Author’s Institution ruled that no formal ethics approval was required in this particular case.

According to our internal procedure the inclusion criteria for donors were: healthy mothers within 15 days to 12 months from delivery. The exclusion criteria were listed in the Italian Association of Human Milk Banks Guidelines [15].

DHM was expressed manually or using an electric milk pump. Sterile polypropylene bottles were used to store the milk, collected within 24 h, at the donors’ homes. All donor milk samples were frozen in a freezer at − 20 °C at the donor’s home, and the temperature was monitored by a thermometer provided by the Human Milk Bank of the author’s Institution. A refrigerated transport assured the cold chain of milk from the donors’ homes to the milk bank. According to the Italian guidelines for the Human Milk Bank, the time between the date of milk collection and the pasteurization cannot exceed 3 months.

DHM was selected and thawed overnight in a refrigerator. Subsequently, the milk bottles were heated to 40 °C using a thermostatic bath to allow proper homogenization of the samples. After that, the milk bottles were stirred and pooled to create pools of multiple donors.

All procedural steps required strict adherence to aseptic techniques, achieved by the use of a biological safety cabinet.

Three raw milk aliquots of 10 ml were picked up from each DHM pool, and one of these aliquots was reserved for the microbiological safety check after pasteurization. The other two samples were analysed, one before and the other one after pasteurization, for macronutrient content. The protein, lactose and lipids content of the DHM pool were measured using mid-infrared spectroscopy, which is a certified method for human milk analyses. The human milk analyser (HMA, Miris AB®, Uppsala, Sweden) was designed for the determination of physiological variation in human milk’s macronutrients. The instrument was calibrated for breast milk measurements using the Kjeldahl method for protein, HPLC for lactose and the Rose – Gottlieb method for fat. The HMA was used in the mode for processed milk [16]. Analyses were conducted on 10-ml samples of each pool, which were previously homogenized using an ultrasonic homogenizer (Sonicator®, Uppsala, Sweden) at 1.5 s/ml, to guarantee the best solubilization of fat and the breakage of casein micelles. These samples were divided into three 3.3 ml aliquots which were analysed using HMA.

In order to obtain reliable results the temperature of all samples was controlled by using a digital probe thermometer. The temperature was maintained between 35 to 40 °C, as recommended by human milk analyser producer.

The analyses show the content of protein, lactose and lipids (g/100 ml) and energy (kcal/100 ml), which is calculated as follows: (9.25 Kcal/g x fat g/100 ml) + (4.40 Kcal/g x protein g/100 ml) + (3.95 Kcal/g x lactose g/100 ml). Three measurements were conducted for each sample, and the mean values for macronutrients and energy were considered. DHM pools were pasteurized by a human milk pasteurizer (S90 Eco sterifeed pasteurizer, Medicare Colgate Ltd., England) by the Holder method (62.5 °C for 30 min), complying with international donor human milk banks guidelines [7–9].

### Statistical analysis

Descriptive data for protein, lipids, lactose and energy are reported as the means and standard deviations (mean ± SD), being data with normal distribution. Macronutrient content before and after HoP were compared using paired t-tests.

Variations in protein, lipids, lactose and energy were also calculated as percent decreases (Delta%), which represent the ratio between the difference in macronutrients before and after pasteurization and the value of macronutrients before pasteurization. In order to evaluate any differences among the decrease of protein, lipids and lactose t-test analysis was performed.

A value of *p* < 0.05 was considered significant. All statistical analyses were performed using SPSS software (SPSS, version 20; SPSS, Chicago, IL).

## Results

A total of 191 donor mothers were enrolled between April 2016 and April 2018 in the authors’ Human Milk Bank of Mangiagalli Clinic in Milan, Italy. Informed consent was obtained from all mothers. The mean age of donors included in the study was 35.01 ± 4.23 years. Their mean duration of gestation was 38.51 ± 2.73 weeks, distributed as follows: 4.0% delivered before 32 weeks, 5.1% between 32 and 36 weeks and 90.9% after 37 weeks. The beginning of donation occurred at 2.9 ± 2.3 months after delivery.

The macronutrient composition of 460 pools was evaluated before and after HoP. Pools had a mean volume of 1607.78 ± 644 ml and each pool contained contributions from 3.5 ± 1.7 mothers.

The volume of milk derived from the different donors was balanced equally for each pool.

The mean macronutrients content before and after pasteurization are described in Table 1.

**Table 1:** Macronutrient concentration before and after pasteurization (mean ± SD)

	Before pasteurization	After pasteurization	*p* value
Protein (g/100 ml)	0.88 ± 0.20	0.86 ± 0.20	< 0.0001
Lactose (g/100 ml)	7.19 ± 0.41	7.11 ± 0.48	< 0.0001
Lipids (g/100 ml)	2.91 ± 0.89	2.75 ± 0.84	< 0.0001
Energy (kcal/100 ml)	60.99 ± 8.10	59.38 ± 7.81	< 0.0001

As shown in Table 1, the concentration of all macronutrient was decreased significantly after the pasteurization process.

**Fig. 1:**
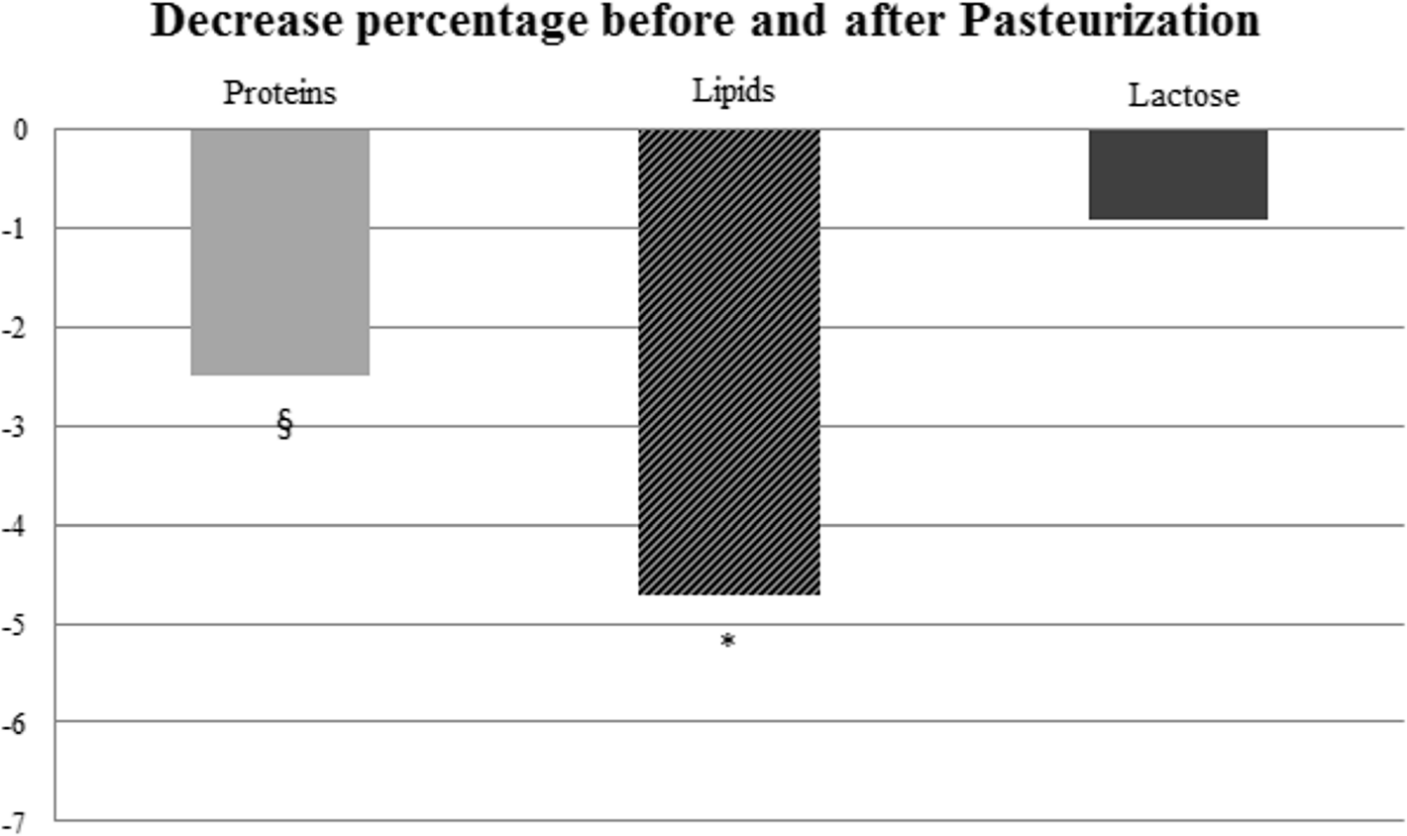
Decreased percentages of macronutrients after pasteurization. *: *p* < 0.001 Lipids vs protein and lactose Delta%; §: *p* = 0.001 Protein vs lactose Delta%

The mean Delta% was − 2.51 ± 13.12% for protein; − 0.92 ± 5.92% for lactose, − 4.79 ± 9.47% for lipids and − 2.48 ± 5.19 for energy. Among all macronutrients, lipids and protein were the most decreased macronutrient after pasteurization, while lactose demonstrated a marginal decrease (Fig. 1).

## Discussion

The present study shows that HoP significantly reduces the macronutrient content of DHM. Lipids and protein are the most affected components, while lactose is slightly reduced. Therefore, the decrease in energy content is mainly due to the decrease in lipid content.

Several studies have shown significant changes of macronutrient content of DHM after pasteurization. Vieira et al., in their analysis of 57 raw human milk samples, found significant reductions in fat and protein mean concentrations following pasteurization (5.5 and 3.9%, respectively) [17]. García-Lara et al., in a total of 34 samples of frozen breast milk, observed significant reductions in fat (3.5%) and energy content (2.8%) after pasteurization, but no significant changes in nitrogen content [18].

Regarding lactose, a recent review showed that, even using different analytical techniques, carbohydrates are not significantly affected by HoP [13].

Freezing, thawing and pasteurization processes may decrease the overall antioxidant capacity of milk [18]. This occurrence and the adherence of human milk fat to the bottle wall could even increase the loss of lipids [19].

In order to limit the residual nutrients attached to the walls of the containers, all milk bottles were heated to 40 °C using a thermostatic bath to allow proper homogenization of the samples. In addition, to avoid possible bias on determining macronutrient content, the same type of polypropylene containers were used to collect and store milk for all donors before and after pasteurization.

It is well known that pasteurization affects bioactive proteins, that are higher in colostrum compared to mature milk [13–20]. It school be noted that in the study, as recommended in the author’s internal procedure, were included donors at least 15 day after delivery and the mean postnatal age at the beginning of donation was 2.9 months of life. Consequently all pasteurized milk was mature. In addition the use of pooled milk has the advantage that it includes milk from donors in different stages of lactation.

Due to its well-known beneficial effect of preventing comorbidities in preterm infants, DHM is the best alternative to the mother’s own milk when compared with formula feeding [21, 22]. However, the DHM changes after HoP have clinical consequences that represent a major challenge to ensuring adequate postnatal growth. Indeed, in order to achieve the high nutritional needs of preterm infants [23], it is mandatory to perform a fortification that takes into account the changes in macronutrient content due to HoP. A tailored approach to fortification of DHM, considering the slight reduction of lactose after HoP compared with lipids and protein, will not lead to an excessive increase in the osmolarity, without a consequent change in gastrointestinal intolerance. Indeed, although the current guidelines are not based on solid scientific evidence, it is recommended that the osmolarity not exceed 450 mOsm/kg (i.e., an osmolarity of 400 mOsm/L) to reduce gastrointestinal tolerance and the risk of necrotizing enterocolitis [24].

It’s well known that HoP affects the bile salt-stimulated lipase (BSSL), which is responsible for the digestion and absorption of fat [17]. In a recent randomized controlled trial performed with preterm infants, it is reported that the inactivation of the BSSL did not affect the gastric digestion of lipids and that the total amount of fat free acids released from milk triglycerides between 35 to 90 min after gastric digestion it was not changed by HoP. However, the BSSL can influence the fat intestinal digestion, especially for preterm infants, that have an immature human pancreatic lipase [25].

Despite the fact that HoP is actually the recommended method in all international human milk bank guidelines [7–9], there is a need to investigate other heating processes capable of treating DHM for ensuring microbiological safety while preserving the milk’s bioactive and nutritive components. Baro et al. investigated the different effect of HoP and High-Temperature-Short-Time pasteurization (HTST) on the human milk protein profile [26]. In addition, in a recent review, Peila et al. have investigated processing techniques other than HoP, focusing in particular on High Pressure Processing, HTST, ultraviolet irradiation and ultrasonic processing. The authors concluded that data on the microbiological safety and on the improvement of the nutritional quality and bioactivity of milk pasteurized by these novel technologies are still scarce [27].

It is known that the use of a tailored fortified human milk is preferred to ensure an adequate postnatal growth of preterm infants [28, 29]. In this study it was demonstrated that HoP leads to macronutrient reduction of human milk. This reduction, although significant, is clinically quite irrelevant, above all since the DHM should be fortified because it is usually mature milk donated by women who delivered full term children.

## Conclusions

This study demonstrated that Holder pasteurization modifies the macronutrient content of donor human milk, particularly reducing the content of lipids and protein.

## Abbreviations

DHM: Donor human milk
HMA: Human milk analyzer
HoP: Holder pasteurization
